# Tumor microenvironment-responsive spherical nucleic acid nanoparticles for enhanced chemo-immunotherapy

**DOI:** 10.1186/s12951-023-01916-0

**Published:** 2023-05-26

**Authors:** Bing Ma, Yingying Ma, Bo Deng, Pengjun Xiao, Pengyu Huang, Dali Wang, Lanxia Liu

**Affiliations:** 1 Tianjin Key Laboratory of Biomedical Materials, Key Laboratory of Biomaterials and Nanotechnology for Cancer Immunotherapy, Institute of Biomedical Engineering, Chinese Academy of Medical Sciences & Peking Union Medical College, Tianjin, 300192 People’s Republic of China; 2 School of Chemistry and Chemical Engineering, Shanghai Jiao Tong University, 800 Dongchuan Road, Shanghai, 200240 People’s Republic of China; 3 Zhangjiang Institute for Advanced Study, Shanghai Jiao Tong University, Shanghai, 201203, People’s Republic of China

**Keywords:** Nanoparticle, Self-assembly, Immunotherapy, Anti-tumor immunity, Combination therapy

## Abstract

Certain chemotherapeutics can induce tumor cells’ immunogenic cell death (ICD), release tumor antigens, and thereby trigger personalized antitumor immune responses. Co-delivery of adjuvants using nanocarriers could amplify the ICD-induced tumor-specific immunity achieving a synergistic chemo-immunotherapeutic effect. However, complicated preparation, low drug loading efficiency, and potential carrier-associated toxicity are the major challenges that limited its clinical applications. Herein, a carrier-free core–shell nanoparticle (MPLA-CpG-sMMP9-DOX, MCMD NPs) was constructed by facile self-assembly of spherical nucleic acids (SNA) with two adjuvants of CpG ODN and monophosphoryl lipid A (MPLA) as a core and doxorubicin (DOX) radially around the dual-adjuvants SNA as a shell. The results demonstrated that MCMD NPs could enhance drugs accumulation in tumors, and release DOX upon enzymatic degradation of matrix metalloproteinase-9 (MMP-9) peptide in the tumor microenvironment (TME), which enhanced the direct-killing effect of DOX on tumor cells. The core of MPLA-CpG SNA efficiently boosted the ICD-induced antitumor immune response to further attack tumor cells. Thus, MCMD NPs achieved a synergistic therapeutic effect of chemo-immunotherapy with reduced off-target toxicity. This study provided an efficient strategy for the development of a carrier-free nano-delivery system for enhanced cancer chemo-immunotherapy.

**Supplementary Information:**

The online version contains supplementary material available at 10.1186/s12951-023-01916-0.

## Introduction

Tumor immunotherapy revolutionizes cancer treatments and has been one effective anti-tumor method [1]. Tumor immunotherapy targets and revives the immune system to recognize and then attack tumor cells [2, 3]. It has been proven that immunotherapy has achieved notable clinical success in the treatment of certain tumors [4]. However, limited efficacy and/or significant toxicities are still the major challenges restricting its broad application [5]. Therefore, it’s still urgent to develop new immunotherapeutic approaches or combinatorial regimens to increase therapeutic efficacy.

Therapeutic tumor vaccines which can elicit an antigen-specific immune cascade against cancers have emerged as an attractive approach in the cancer immunotherapy field [6]. However, the lack of tumor-specific antigens is the major obstacle in the development of therapeutic tumor vaccines. Some studies demonstrated that certain chemotherapeutic agents (e.g., DOX) could induce ICD, release tumor antigens as well as high mobility group box 1 (HMGB1), and then recruit and activate antigen-specific immune cells eliciting personalized antitumor immune responses at local tumor tissue, which was also named in situ vaccine [7, 8]. Normally, the immune response was not potent enough to eliminate the tumor cells. Accumulating evidence suggested that co-delivery of adjuvants and chemotherapeutic agents using nanomaterials could amplify the ICD-induced immunity as well as reduce the risk of cytokine storm of adjuvants and severe off-target toxicity of chemotherapeutic agents after systemic administration [9–11]. Nanoparticles possess lots of unique advantages over traditional drugs [12], such as protecting antigens and adjuvants against enzymic degradation, navigating complex physical barriers, targeted delivery to specific cells, and improving delivery efficiency [13, 14]. However, most nanoparticles prepared with nanomaterials were difficult to ensure drug loading efficiency and precise administration [15, 16]. Additionally, complicated preparation and potential toxicity also limited the clinical applications [17].

**Scheme 1:**
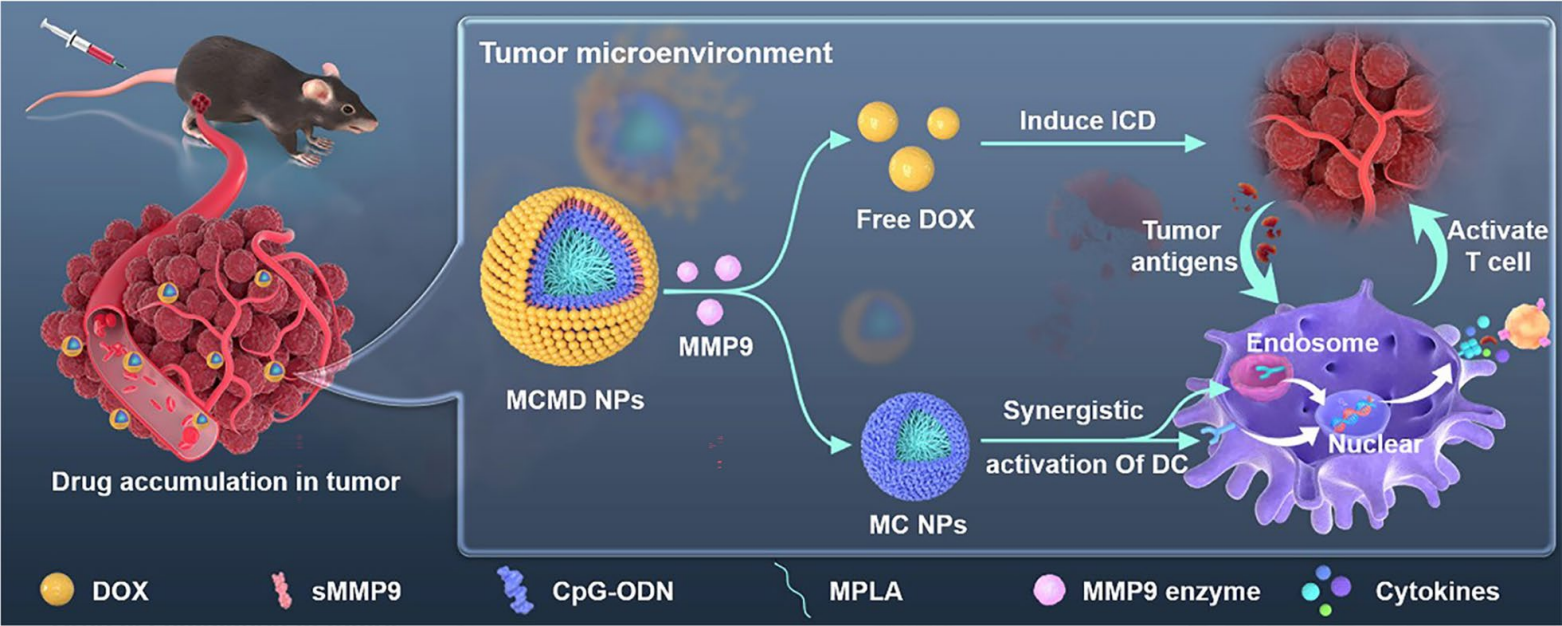
Schematic illustration of the synergistic anti-tumor chemo-immunotherapeutic mechanism of carrier-free MCMD nanoparticles

Spherical nucleic acids (SNAs) with a shell of densely oriented oligonucleotides around a core could protect the nucleic acids against nuclease and promote their rapid endocytosis into DCs via receptor-mediated pathways, showing a great application prospect in medical sciences [18, 19]. Herein, we designed carrier-free nanoparticles using MPLA-CpG SNA (MC NPs) as a core and arranged chemotherapeutic drug DOX radially around the SNA particle forming a spherical shell of DOX to spatiotemporally deliver DOX and adjuvants MPLA and CpG. DOX was linked on the adjuvants-core with a substrate peptide of matrix metalloproteinase 9 (sMMP9), a TME-responsive peptide, to ensure that DOX could effectively release in TME to kill tumor cells and simultaneously generate tumor-specific antigens upon the nanoparticles reached in tumor tissue, meanwhile, the MPLA-CpG SNA core could high-efficiently boost the ICD-induced tumor-specific personalized immune response to further attack tumor cells improving the antitumor efficacy (Scheme 1). This study aims to enhance drug accumulation in tumor tissue and achieved a synergistic therapeutic effect of chemo-immunotherapy with reduced off-target toxicity.

## Results and discussion

### Characterization of MPLA-CpG-sMMP9-DOX

**Fig. 1:**
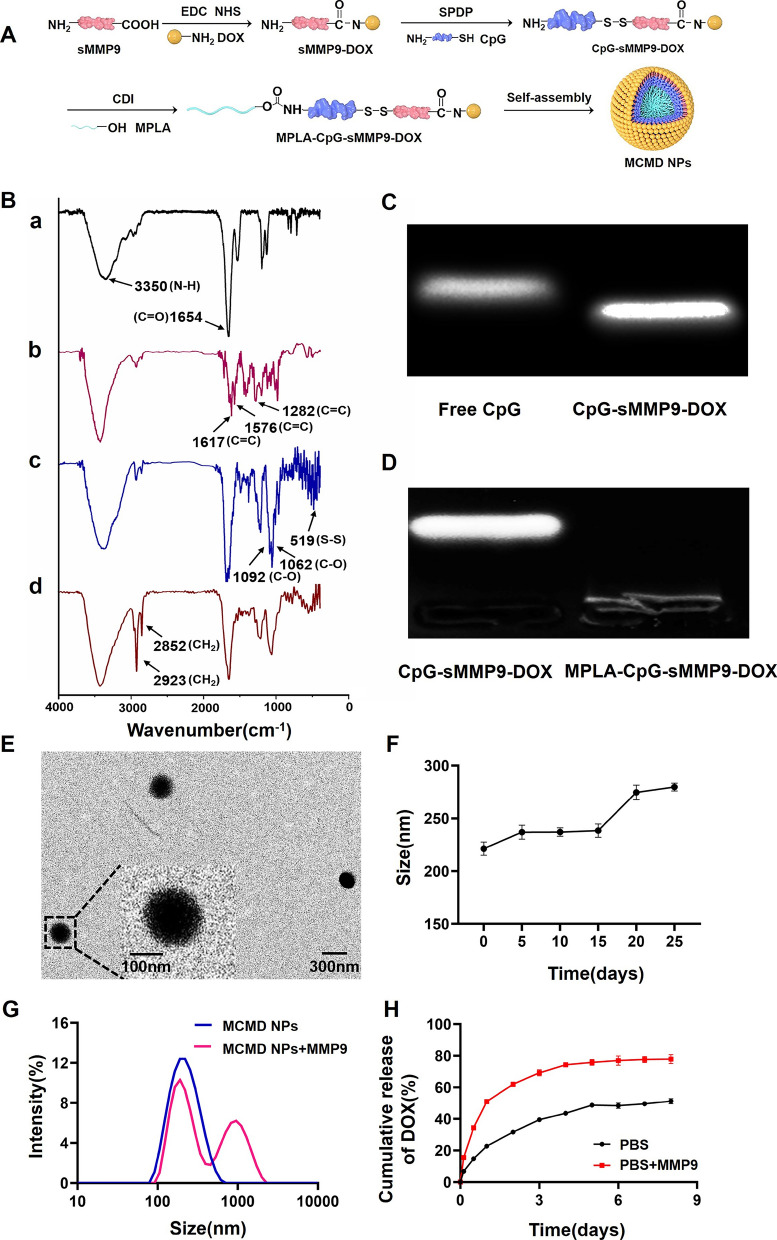
Characterization of MCMD NPs. **A** The detailed synthesis route of MCMD NPs. **B** The FITR of MCMD (a: sMMP9, b: sMMP9-DOX, c: CpG- sMMP9-DOX, d: MPLA-CpG-sMMP9-DOX). **C** The agarose gel electrophoresis of free CpG and CpG-sMMP9-DOX. **D** The agarose gel electrophoresis of CpG-sMMP9-DOX and MPLA-CpG-sMMP9-DOX. **E** The typical TEM image of MCMD NPs. **F** The size distribution of MCMD NPs in PBS. **G** The size distribution of MCMD NPs against the MMP9 enzyme or not. **H** The DOX release profile from MCMD NPs with MMP9 enzyme or not. Data were expressed in the form of mean ± SD (n = 3)

To ensure the accurate loading of chemotherapeutic agents and adjuvants and fabricate the nanoparticles by self-assembly, we synthesized an amphiphilic molecule of MPLA-CpG-sMMP9-DOX via three steps reaction (Fig. 1A). First, DOX was covalently bound to sMMP9 through the reaction of an N-hydroxysuccinimide (NHS) ester and amines. Then, the resulting sMMP9-DOX was activated with SPDP and linked with sulfhydryl-modified CpG ODN to yield CpG-sMMP9-DOX conjugate through a disulfide exchange reaction. Lastly, MPLA was conjugated with CpG-sMMP9-DOX through an amidation reaction to yield the MPLA-CpG-sMMP9-DOX [20]. To confirm the successful synthesis, the purified conjugates of each step were characterized with Fourier‐transform infrared spectroscopy (FTIR). As shown in Fig. 1B-a and b, compared to sMMP9, newly appeared stretching vibration peaks of the benzene ring skeleton (1617.7 cm^−1^, 1576.9 cm^−1^, and 1282.7 cm^−1^) in sMMP9-DOX proved that DOX was successfully conjugated with sMMP9. In Fig. 1B-b, 1092.5 cm^−1^ and 1062.4 cm^−1^ were C–O stretching vibration peaks of deoxyribose in CpG, and the absorption peak at 519.8 cm^−1^ indicated that CpG and sMMP9 are successfully connected via disulfide bond generating CpG-sMMP9-DOX conjugate. In Fig. 1B-d, the methylene characteristic peaks (2923.1 cm^−1^ and 2852.6 cm^−1^) belonging to MPLA indicated that the amphiphilic molecule MPLA-CpG-sMMP9-DOX (MCMD) was successfully synthesized. The results of Gel retardation of CpG-sMMP9-DOX and MPLA-CpG-sMMP9-DOX further confirmed the successful synthesis of MPLA-CpG-sMMP9-DOX (Fig. 1C and D).

### MCMD NPs preparation and characterization

Amphiphilic MCMD has dimensionless packing parameter P according to Israelachvili's surfactant theory. The “P” value of amphiphilic molecules is related to the morphology of these molecules self-assembled, and when P < 1/3, spherical morphologies are produced. In this study, the “P” value of MCMD was 0.325, which suggests that these molecules were inclined to self-assemble into a spherical structure [21]. TEM results showed that MCMD assemblies in water did display spherical morphology with uniform distribution and good dispersibility (Fig. 1E). The size of MCMD nanoparticles (MCMD NPs) in aqueous solution was measured by DLS with a diameter of 159.3 ± 0.8 nm and the polydispersity Index of 0.26 ± 0.02 (Additional file 1: Fig. S1). Zeta potential measurements indicate that the MCMD NPs have a negative charge (− 36.5 ± 0.2 mV) in pure water (Additional file 1: Table S1). To investigate the stability of MCMD NPs, they were kept in PBS for 25 days at 4 ℃. The size change of MCMD NPs was monitored using the DLS. It is found that no momentous change in the diameter of MCMD NPs is observed over 15 days whereas the apparent change occurred after 15 days (Fig. 1F), demonstrating relatively good stability within 15 days.

To assess whether MCMD NPs could release DOX, MCMD NPs were co-incubated with the MMP9 enzyme (0.2 µg/mL) [22]. Then the size of nanoparticles was measured with DLS, and the release amount of DOX in the dialysate was detected by UV–Vis at preset timepoints. DLS data displayed that the nanoparticles aggregated, and their size increased after co-incubating with the MMP9 enzyme for 4 days (Fig. 1G). The release results showed that the cumulative release amount of DOX in the MMP9 enzyme-added group was up to 76.5 ± 2.8% in the first 4 days, which was much faster than that of the enzyme-free group (47.0 ± 5.7%) (Fig. 1H), indicating that sMMP9 was cleaved by MMP9 enzyme which led to the release of DOX from MCMD NPs. These results suggested that MCMD NPs response to the MMP9 enzyme could accelerate the DOX release from the nanoparticles.

### In vitro experiments

#### Cell viability

**Fig. 2:**
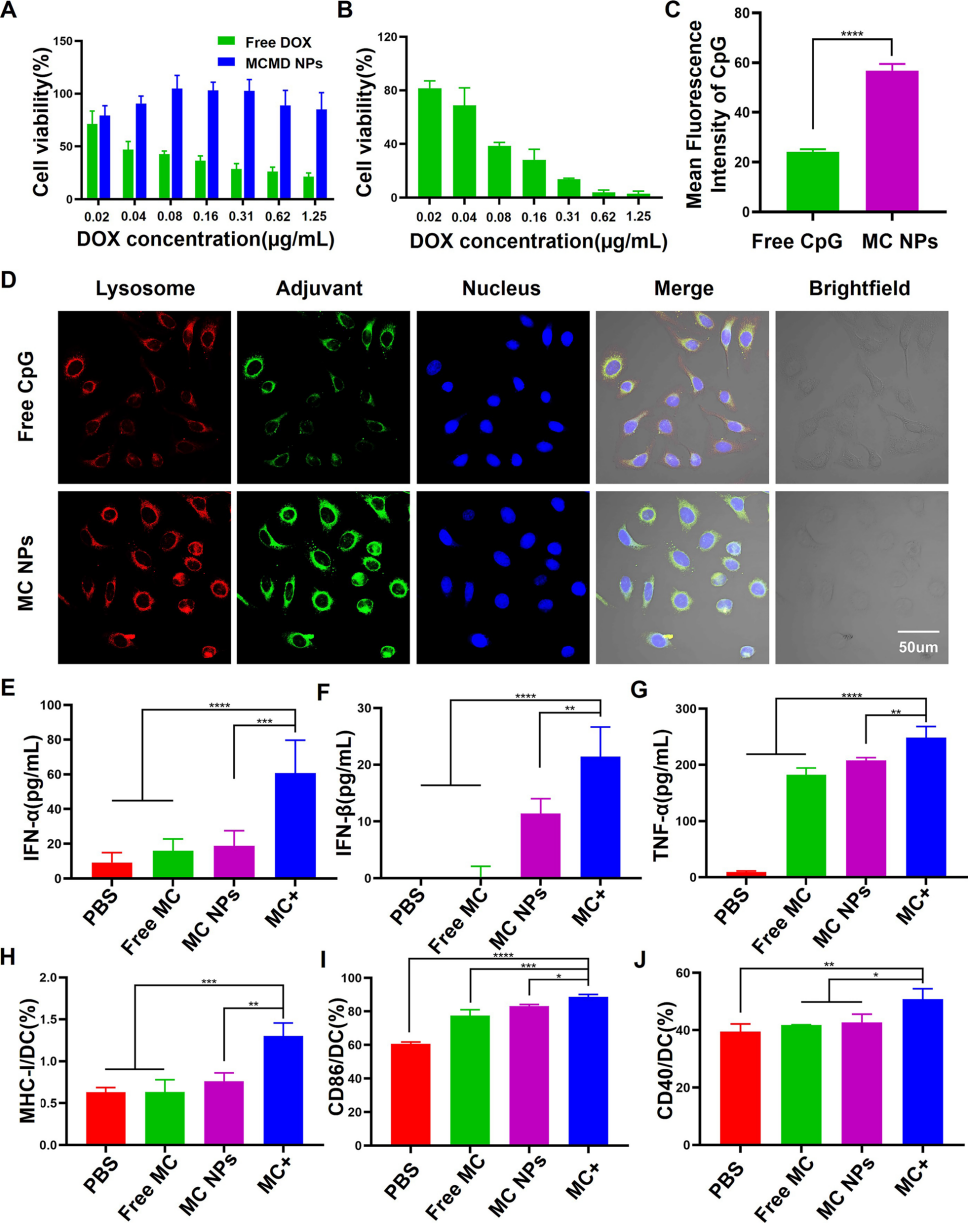
The effect of MCMD NPs on cell viability, DC maturation, and activation. The cell viability of **A** HUVECs and **B** E.G7-OVA treated with free DOX or MCMD NPs. **C** Quantitative analysis of average fluorescence intensity of CpG in DCs. **D** CLSM images of DCs after co-incubation with free CpG or MC NPs. Red: lysosome. Green: Cy5-labeled CpG. Blue: nucleus. The cytokine secretion levels of **E** IFN-α, **F** IFN-β, and **G** TNF-α were measured with ELISA assays. The expression levels of **H** MHC-I, **I** CD86, and **J** CD40 were measured with flow cytometers. Data were expressed in the form of mean ± SD (n = 5). * P < 0.05, ** P < 0.01, ***P < 0.001, ****P < 0.0001

Free DOX was usually administrated by intravenous injection, which results in a high degree of cardiovascular toxicity [23]. To evaluate whether MCMD NPs could decrease the toxic side effects of DOX on normal tissues, human umbilical vein endothelial cells (HUVECs) were incubated with various concentrations of MCMD NPs for 24 h. The cell viability of HUVECs was assessed by CCK-8 assay. HUVECs treated with MCMD NPs showed low cell viability even the DOX concentration reached up to 1.25 µg/mL (Fig. 2A). In contrast, high cytotoxicity was exhibited when the cells were treated with free DOX even at a low concentration of 0.04 µg/mL. Additionally, when MCMD NPs were pretreated with the MMP9 enzyme, it could cause a significant viability decrease of mouse T lymphoma cells (E.G7-OVA) (Fig. 2B).

#### Cellular uptake and DCs activation

We next investigated whether dual-adjuvant MC NPs as the core of MCMD NPs could drive the internalization of adjuvants into dendritic cells (DCs) and stimulate DCs maturation and activation which could initiate a cascade of anti-tumor immune responses. After Cy5-labeled MCMD NPs were pretreated with MMP9 enzyme and ultracentrifuged to remove the supernatant, the remaining nanoparticles were incubated with DCs for 6 h. DCs were imaged with a confocal laser scanning microscope (CLSM) and the fluorescence intensity in cells was quantified using ImageJ software. The results showed that the fluorescent signal in MC NPs group was 2.6-fold stronger than the free CpG group (Fig. 2C and D). Additionally, MC NPs could increase the expressions of MHC-II and CD80 molecules on the surface of BMDCs which could present antigens to T cells (Additional file 1: Figs. S2 and S3). Meanwhile, the results of enzyme-linked immunosorbent assay (ELISA) demonstrated that MC NPs could significantly stimulate DCs secretion of TNF-α and Type I interferon (including IFN-α and IFN-β) compared with free adjuvants and PBS (Fig. 2E–G). TNF-α can induce immature DC maturation and differentiation. IFN-α and IFN-β can activate T, B, and natural killer cells (NK cells) against tumor cells [24].

To mimic the TME and further study whether MCMD NPs could efficiently promote BMDCs to present tumor antigens in tumor tissue, BMDCs were incubated with MC NPs and tumor cell fragments obtained from treated E.G7 tumor cells with DOX, which was named MC + group, and then evaluated the expression level of MHC-I/II and co-stimulated molecules. The results showed that not only the expression level of CD40 but also MHC-I and CD86 on DCs were up-regulated (Fig. 2 H-J), indicating that MCMD NPs could promote tumor antigens presented via the MHC-II pathway to activate CD4^+^ T cells as well as cross-presented via the MHC-I pathway to activate CD8^+^ T cells [25].

### In vivo experiments

#### Biodistribution

**Fig. 3:**
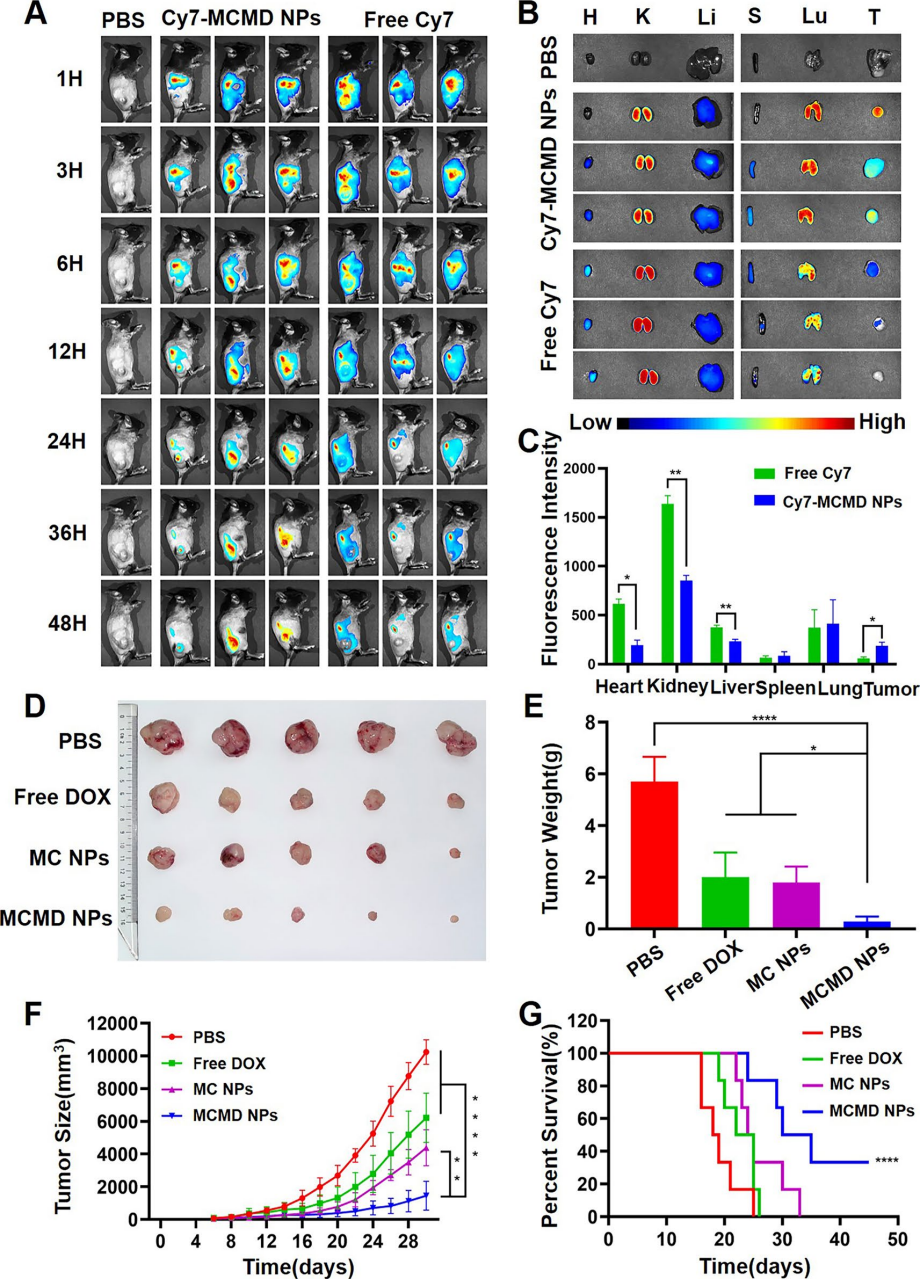
Biodistribution and therapeutic effect of MCMD NPs. **A** The images of free Cy7 and Cy7-MCMD NPs accumulated at the tumor site at selected time-points. **B** The fluorescence images of tumors and organs from mice at 48 h after injection. **C** Average fluorescence intensity of organs and tumors in each group. **D** Tumor images. **E** Average tumor weight of each group. **F** The curves of tumor growth of tumor-bearing mice. **G** The survival curves of tumor-bearing mice. Data were expressed in the form of mean ± SD (n = 5). *P < 0.05, **P < 0.01, ****P < 0.0001

Nanoparticles are expected to prolong the blood circulation of drugs and enhance their tumor-targeting capability [26, 27]. To study the biodistribution of MCMD NPs, Cy7 labeled MCMD NPs (Cy7-MCMD NPs) were injected into C57BL/6 mice bearing subcutaneous E.G7 OVA xenografts through the tail vein. The Cy7 fluorescence in the mouse model was detected by in vivo imaging system (CRI Maestro EX, USA). Free Cy7 rapidly accumulated at the tumor site in the first 3 h but rapidly disappeared after 24 h, whereas Cy7-MCMD NPs gradually accumulated in tumors and showed a strong fluorescence signal even after 48 h (Fig. 3A). These results indicated that MCMD NPs could increase the accumulation of DOX and adjuvants in tumors.

To further observe the biodistribution and clearance of the drug, main organs, and tumors were collected after 48 h post administration and imaged by CRI. Compared to free Cy7, Cy7-MCMD NPs remarkably decreased the amount of Cy7 fluorescence in the kidney (Fig. 3B and C), indicating that these nanoparticles could hinder renal excretion. This can be attributed that renal excretion of molecules or nanoparticles was size-dependent and the nanoparticles larger than 30 nm could diminish the clearance through kidneys [28]. Of note, despite the fact that MCMD NPs decreased the renal accumuation to a greater extents compared to free DOX, the possibility of sustained and slow release of DOX might lead to potential renal toxicity [29, 30]. To prevent the DOX-induced renal toxicity, some protective adjuvants like atorvastatin and/or theanine may be adopted as a component against DOX toxicity, via antioxidant, anti-nitrosative, anti-inflammatory and anti-apoptotic mechanisms [31, 32]. Additionally, MCMD NPs significantly enhanced drug accumulation in tumors to 3.2-fold higher compared to free Cy7, likely due to the enhanced permeability and retention (EPR) effect. On the other hand, Cy7-MCMD NPs significantly decreased drug accumulation in the heart compared with free Cy7, suggesting that the nanoparticles might attenuate DOX-induced cardiotoxicity which is the most noteworthy side effect of DOX [33]. However, many MCMD nanoparticles were still located in the lung, showing no significant selectivity between tumors and the lung. To further achieve a better targeting capability, the nanoparticles should be modified with tumor-specific antibodies or aptamers with high binding affinity [34, 35]. Collectively, these results showed that MCMD NPs could promote drug accumulation at the tumor site and decrease heart accumulation as well.

#### Therapeutic effect of MCMD NPs

To further assess the in vivo therapeutic effect of MCMD NPs, we established an E.G7-OVA tumor model using C57BL/6 mice. The tumor size was measured regularly to draw a tumor growth curve after mice were treated with various formulations. When the tumor volume exceeded 2000 mm^3^, the mice were regarded as dead. The results showed that free DOX and MC NPs inhibited tumor growth to some extent compared with PBS, while MCMD NPs exhibited the most powerful capability of retarding tumor growth, even though some tumors almost completely stop growing. The average tumor weight of the mice treated with MCMD NPs was 0.28 ± 0.19 g, which was much smaller than that of the PBS group (5.70 ± 0.95 g), the free DOX group (and 2.00 ± 0.94 g) and the MC NPs group (1.79 ± 0.62 g) (Fig. 3D–F). However, the body weight of the mice treated with MCMD NPs was not affected during the treatment process, demonstrating the biological safety of MCMD NPs (Additional file 1: Fig. S4).

Meanwhile, the results of the survival period showed a similar trend to the tumor volume curve. The mice treated with PBS gradually died from 16 to 25 days, while free DOX prolonged the survival time slightly, and the MC NPs group prolonged the survival period moderately. In contrast, MCMD NPs significantly prolonged the survival time of tumor-bearing mice, with 33% of the mice surviving more than 45 days (Fig. 3G).

The hematoxylin–eosin (H&E) staining results demonstrated that, compared with other groups, MCMD NPs induced a larger area of typical apoptosis in tumor tissues, such as a reduction in cell size, separation from surrounding cells, nucleus concentration, and nuclear karyoplasm [36]. Moreover, the pathological analysis of heart tissue also showed that MCMD NPs effectively reduced cardiotoxicity compared to free DOX (Additional file 1: Fig. S5). These results were consistent with the previous study which demonstrated that nanoparticle-based chemotherapy was superior to conventional DOX chemotherapy in terms of reduction in the risk of cardiotoxicity [37, 38].

The above in vivo results showed that MCMD NPs had a good capability to enhance drug accumulation in tumors, induce tumor apoptosis, and then inhibit tumor growth, and significantly prolonged the survival period with reduced side toxicity.

#### Antitumor immune responses in vivo

**Fig. 4:**
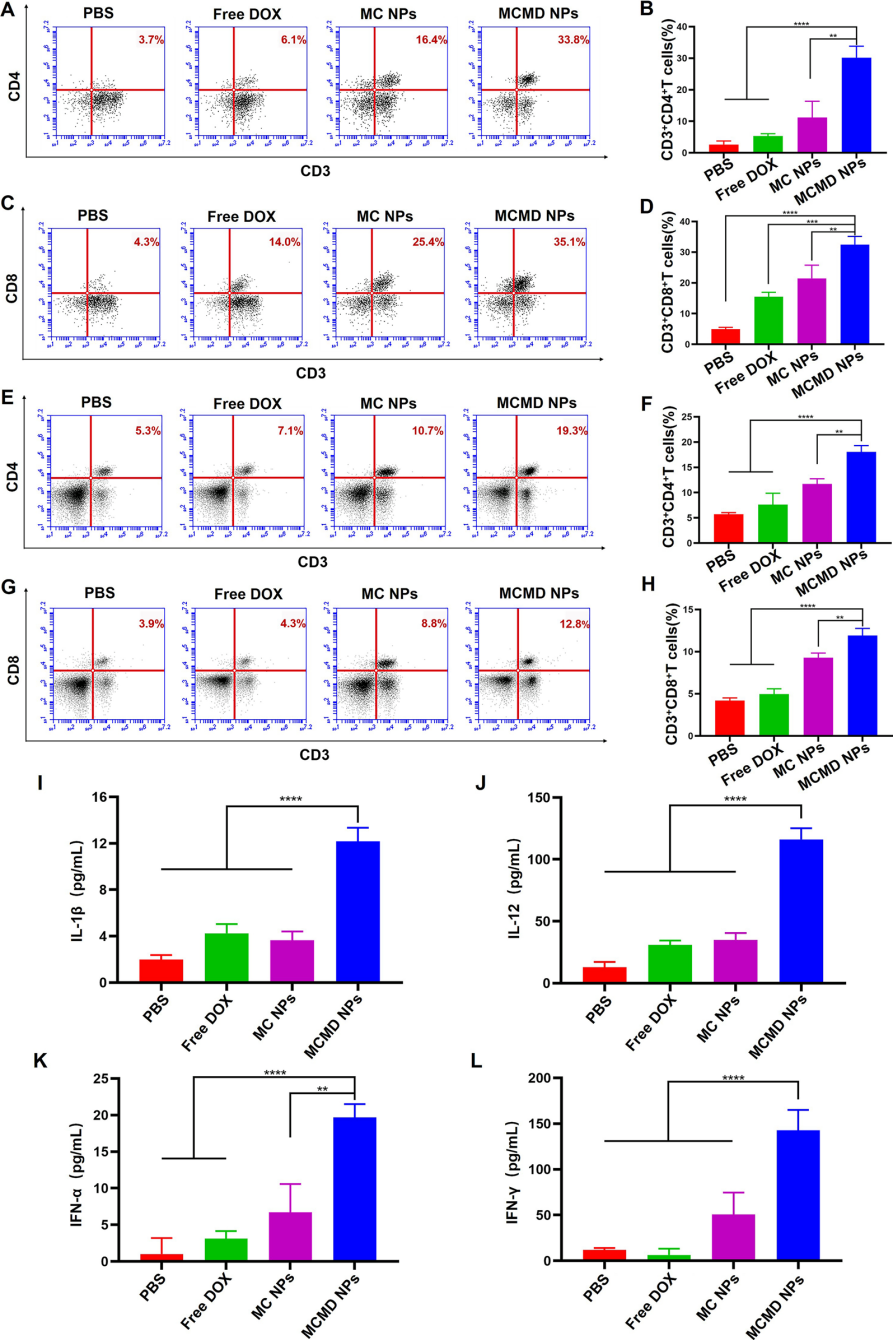
MCMD NPs significantly enhanced the antitumor immune responses in vivo. Representative FACS plots and histogram of percentage of CD3^+^ CD4^+^ and CD3^+^ CD8^+^ T cells in **A**–**D** tumors (**A**, **B**: CD3^+^CD4^+^; **C**, **D**: CD3^+^CD8^+^) and **E**–**H** spleens (**E**, **F**: CD3^+^CD4^+^; **G**, **H**: CD3^+^CD8^+^). Cytokines **I** IL-1β, **J** IL-12, **K** IFN-α, and **L** IFN-γ were evaluated with ELISA kits. Data were expressed in the form of mean ± SD (n = 5). * P < 0.05, ** P < 0.01, ***P < 0.001, ****P < 0.0001

To further explore whether MCMD NPs could efficiently enhance the local anti-tumor immune response in tumor tissue and stimulate systemic immunity. The immune levels in tumors, lymph nodes, and spleens collected from mice treated with various formulations were evaluated. The results revealed that not only the proportion of CD4^+^ T cells in tumor tissues treated with MCMD NPs was about ninefold, fivefold, and twofold compared to PBS, free DOX, and MC NPs, respectively, but also the proportion of CD8^+^ T cells was eightfold, 2.5-fold and 1.4-fold compared with the three control groups, respectively (Fig. 4A–D). The results suggested that MCMD could significantly amplify the DOX-induced tumor-specific immune responses. T cell expansion was important in overcoming the suppressive TME and yielding long-term improved therapeutic outcomes [39]. Besides within the tumor tissue, MCMD NPs also significantly increased the proportion of T cells in the adjacent lymph nodes. The proportion of CD4^+^ and CD8^+^ T cells in lymph nodes increased over fourfold in MCMD NPs group compared with the PBS group. The percentage of CD4^+^ T cells increased from 10.9% to 46.8%, and CD8^+^ T cells increased from 8.8 to 37.1% (Additional file 1: Fig. S6). Moreover, MCMD NPs increased the percentage of CD4^+^ T cells even in the spleens from 5.3 to 19.3%, and CD8^+^ T cells from 3.9 to 12.8% which both were close to fourfold compared to the PBS group (Fig. 4E–H). Therefore, the nanoparticles could induce synergistic anti-tumor immune responses in terms of T cell proliferation. These results indicated that MCMD NPs could not only enhance the anti-tumor immune responses at tumor tissue leading to the local tumor destruction but also could stimulate the systemic immune responses leading to the distant lesion of metastatic tumors.

Cytokines are derived from activated immune cells and are involved in the whole process of anti-tumor immune responses, including antigen presentation, immune cell interaction and activation. Cytokines were related to the type and intensity of immune responses. DOX-triggered ICD could activate the pyrin domain containing-3 protein (NLRP3)-dependent (inflammasome) pathway releasing some cytokines, such as IL-1β [40, 41]. Our previous study demonstrated that MC NPs could synergistically activate TLR4 and TLR9 signaling pathways and generate robust Th1-biased immune responses, releasing IL-12 and interferons [42]. So, splenic lymphocytes collected from mice treated with various formulations were co-cultured with DOX-treated E.G7 cell debris for 72 h and the supernatants were collected to detect cytokines of IL-1β, IL-12, IFN-α and IFN-γ. As shown in Fig. 4I–J, the level of IL-1β was enhanced by about 6.0-, 2.9-, and 3.4-fold compared with PBS, free DOX, and MC NPs. IL-12 was enhanced about 8.9-, 3.4-, and 3.3-fold compared with PBS, free DOX, and MC NPs. The level of IFN-α secreted by splenic lymphocytes treated with MCMD NPs was 19.6-, 6.3-, and 2.9-fold higher than those of PBS, free DOX, and MC NPs (Fig. 4K). IFN-α can not only regulate the innate immune responses by promoting the function of antigen-presenting cells (APC) and NK cells but also activate the adaptive immune system by promoting antigen-specific T and B cell immune responses and immunological memory [43]. In addition, the level of IFN-γ treated with MCMD NPs was also significantly increased compared with other groups (Fig. 4L). IFN-γ belongs to type II interferons, which can induce Th1 cell differentiation and promote CD8^+^ cytotoxic T cell response (CTLs). Collectively, these results indicated that MCMD NPs could efficiently induce improved antitumor immune responses.

#### T cell immune memory

**Fig. 5:**
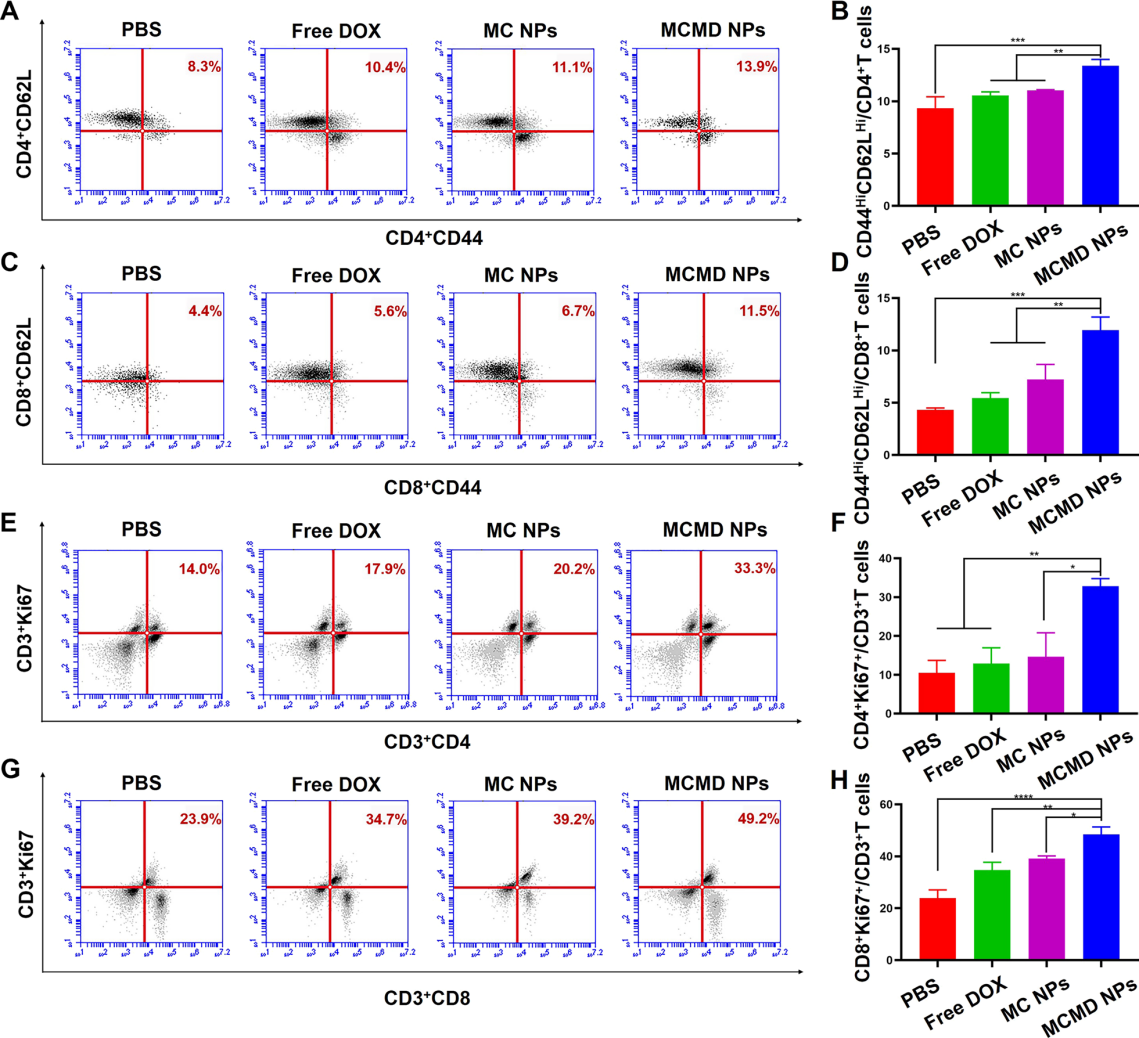
Generation of T cells immune memory. Representative FACS plots and histogram of the percentage of **A**, **B** CD4^+^ and **C**, **D** CD8^+^ T memory cells. The proliferation of **E**, **F** CD4^+^ and **G**, **H** CD8^+^ T cells from mice in different groups after being re-stimulated with tumor debris in vitro. (n = 5). * P < 0.05, ** P < 0.01, ***P < 0.001, ****P < 0.0001

To evaluate whether MCMD NPs could effectively induce the differentiation of memory cells and provide a long-term protective immune response, the spleen lymphocytes from mice treated with various formulations were collected and re-stimulated with E.G7 cell debris in vitro. The proliferation of memory cells including central memory T cells (Tcm) and effector memory T cells (Tem) was detected [44]. The results showed that the percentage of CD4^+^ Tcm and CD8^+^ Tcm in the MCMD NPs group was enhanced about 1.7- and 2.5-fold than the PBS group, respectively (Fig. 5A–D), indicating that MCMD NPs could induce an antigen-specific immune response upon encountering the same tumor-related antigens for a long duration, which meant that the nanoparticles could prevent the tumor recurrence to a certain extent. Additionally, after being re-stimulated with E.G7 cell debris, the expansion of T cells was significantly expanded, the percentages of CD4^+^Ki67^+^ T cells and CD8^+^Ki67^+^ T cells in the MCMD NPs group reached 28.7% and 49.2% respectively, which were significantly higher than those in PBS group (CD4^+^Ki67^+^ T cells were 6.9%, CD8^+^Ki67^+^ T cells were 23.9%) (Fig. 5E–H). The results further confirmed that MCMD NPs could generate memory cells that could rapidly react upon encountering the same antigen again [45]. All the results indicated that MCMD NPs could effectively produce immune memory which plays a role in preventing cancer recurrence and metastasis.

## Conclusions

In summary, we successfully constructed a TME-responsive spherical nucleic acid nanoparticle MCMD NPs with dual-adjuvant MPLA-CpG SNA as a core and DOX radially on its outer layer as a shell. The nanoparticles could ensure the precise loading of chemotherapeutic agents and adjuvants, and enhance drug accumulation at the tumor site. Moreover, MCMD NPs had TME-responsive capability which allowed DOX release to directly kill tumor cells and generated ICD-induced tumor-specific immune response upon MCMD NPs reaching tumors. Meanwhile, the internal MPLA-CpG SNA efficiently amplified the DOX-induced tumor-specific immune response, and increased T cell expansion and cytokines secretion. Thus, the nanoparticles remarkably inhibited tumor growth, prolonged the survival period, and reduced the systemic toxicity of DOX, achieving a synergistic therapeutic effect. Overall, this study provided a feasible strategy for the co-delivery of chemotherapeutics and adjuvants for cancer combined therapy.

## Materials and methods

### Synthesis and characterization of MPLA-CpG-sMMP9-DOX

According to some literature and our previous studies[42, 46–48], the conjugation of MPLA-CpG-sMMP9-DOX was synthesized with DOX, sMMP9 (sequence: GPQGIAGQR, ChinaPeptides, China), CpG ODN (Type C 2395, Sangon Biotech, China) and MPLA (Neosun Pharm, China). To synthesize sMMP9-DOX, the carboxyl groups of sMMP9 were activated with 1-ethyl-3-(3-dimethylaminopropyl) carbodiimide/N-hydroxy-succinamide (EDC/NHS, Aladdin, China) in dimethyl sulfoxide (DMSO) for 4 h at room temperature, and reacted with DOX for 24 h using 4-dimethylaminopyridine (DMAP, Aladdin, China) as a catalyst to get sMMP9-DOX. Next, the resulted sMMP9-DOX was pyridyldithiol-activated by succinimidyl 3-(2-pyridyldithio) propionate (SPDP) in anhydrous dimethylformamide (DMF) for 6 h at room temperature [49], and then reacted with sulfhydryl modified CpG ODN 24 h. After removing the unreacted impurities with dialysis (8-kDa MWCO), the hydrophilic conjugation of CpG-sMMP9-DOX was obtained. MPLA as the hydrophobic end of the amphiphilic molecule of MPLA-CpG-sMMP9-DOX was linked with the hydrophilic molecule of CpG-sMMP9-DOX using N, Nʹ-Carbonyldiimidazole (CDI, Aladdin, China) as crosslinker. The hydroxyl group of MPLA was activated by CDI in DMSO for 2 h, and then reacted with the amino group on CpG ODN of CpG-sMMP9-DOX at the molar ratio of 2:1 for 12 h. Unreacted impurities were removed using a 2-kDa MWCO dialysis tubing. The synthesis of MPLA-CpG-sMMP9-DOX was determined by agarose gel electrophoresis and Fourier Transform Infrared Spectrometer (FTIR, Thermo Fisher Scientific, USA).

### Preparation and characterization of MCMD NPs

The amphiphilic molecules of MPLA-CpG-sMMP9-DOX were used to  prepare MCMD NPs by self-assembly. The size and zeta potential of MCMD NPs were measured with dynamic light scattering (DLS) and Zetasizer Nano ZS (Malvern Instruments Ltd., UK), respectively. The morphology was detected using the transmission electron microscope (TEM, JEOL JEM-100CX-II, Japan). The DOX release behaviors from MCMD NPs in the presence of MMP9 enzyme (0.2 µg/mL) at 37 ℃ were evaluated by detecting the amount of DOX in dialysate after nanoparticles dialysis with 1 kDa MWCO dialysis tubing at preset time points. Meanwhile, the size alteration of nanoparticles was detected with DLS to determine whether the remained dual-adjuvants still maintained nanoparticles after enzymolysis of MCMD NPs by MMP9 enzyme releasing DOX.

### In vitro experiments

#### Cell viability

To assess whether MCMD NPs could effectively reduce the cardiovascular toxicity of DOX, HUVEC were co-cultured with free DOX and MCMD NPs at various concentrations for 24 h, respectively. Then the cell viability was evaluated with Cell Counting Kit-8 (CCK-8, Dojindo Molecular Technologies, Inc., Japan).

To evaluate the effect of MCMD NPs on tumor cells, MCMD NPs were pretreated with MMP9 enzyme for 48 h, and then co-cultured with mouse T lymphoma cells (E.G7-OVA) for 24 h before the cell viability was detected.

#### Cellular uptake

To investigate whether the dual-adjuvant core of MCMD NPs could enhance the uptake of adjuvants in dendritic cells (DCs), CpG ODN was labeled with anthocyanin fluorescent dye Cyanine 5 (Cy5) to prepare Cy5-labeled MCMD NPs. After MCMD NPs were pretreated with MMP9 enzyme and the supernatant was removed after ultracentrifugation, the remaining nanoparticles (MC NPs) were co-incubated with DC 2.4 for 6 h (CpG ODN concentration was 20 µg/mL). DCs were fixed with 4% paraformaldehyde after being washed three times with PBS. The nucleus and lysosome were stained with DAPI and Lyso-Tracker Red, respectively. Then the adjuvants amount in DCs were imaged with CLSM (Zeiss LSM 800, Germany) and analyzed with ImageJ software.

#### BMDCs maturation and activation

The bone marrow-derived dendritic cells (BMDCs) used in the experiment were obtained from the femurs of C57BL/6 female mice. After incubated with complete RPMI 1640 medium containing 10% FBS, 20 ng/mL GM-CSF and 10 ng/mL IL-4 at 37 ℃ for 6 days, the immature BMDCs were collected to estimate the effect of MC NPs on maturation and activation.

BMDCs were co-cultured with free MPLA & CpG ODN (free MC), MC NPs (MCMD NPs treated with MMP9 enzyme), and a mixture of MC NPs and tumor debris (MC +) for 24 h (CpG ODN was 20 µg/mL), respectively. Some BMDCs were stained with Cy5.5-labeled anti-CD11c, FITC-labeled anti-MHC-II, and APC-labeled anti-MHC-I antibodies to detect the molecule MHC-I/II expression level on BMDCs. The others were stained with fluorescence labeled anti-CD11c, anti-CD86, anti-CD40, and anti-CD80 antibodies to evaluate the expression of co-stimulating molecules on BMDCs. The expression levels of the above molecules were evaluated by BD Accuri™ C6 flow cytometer (BD Biosciences, USA).

In the meantime, the cytokines of tumor necrosis factor (TNF-α) and Interferon in the supernatant were detected by ELISA kits to analyze the activation effect of MC NPs on DCs.

### In vivo experiments

#### Establishment of the tumor-bearing mouse model

All animals were treated strictly under the guidelines approved by the Animal Ethical and Welfare Committee of Institute of Radiation Medicine Chinese Academy of Medical Sciences & Peking Union Medical College (Approval No. IRM-DWLL-2021074). 6-week-female C57BL/6 mice were subcutaneously injected with 5 × 10^5^ E.G7-OVA tumor cells at their right hips to build a tumor model of lymphoma-bearing mice.

#### Biodistribution

Fluorescence Cy7 labeled-MCMD NPs (Cy7-MCMD NPs) were prepared by encapsulating Cy7 dye in MCMD NPs. Then the Cy7-MCMD NPs were administrated via tail vein injection till the tumor volume reached about 300 mm^3^. Free Cy7 was used as a control (Cy7 content was 20 µg/mouse). The distribution of fluorescence signals in mice at different time points was inspected by in vivo imaging system (CRI Maestro EX, USA). Furthermore, the organs and tumors were collected to detect the fluorescence intensity for analysis of the drug distribution at 48 h after injection.

#### Therapeutic effect

The mice were treated with PBS, free DOX, MC NPs, and MCMD NPs (n = 5, the content of DOX was 100 µg/mouse), respectively, when the tumor volume reached about 50 mm^3^. All mice were treated 3 times in total with an interval of 5 days. During this period, the size of the tumor and the weight of the mouse were measured every two days. While the tumor volume exceeded 2000 mm^3^, the mouse was judged as dead. The anti-tumor effect of MCMD NPs in vivo was evaluated by drawing tumor growth curves and recording the survival time.

Some mice were treated 3 times and sacrificed 72 h after the last treatment. The lymph nodes, spleens, and tumor tissues of mice were collected for pathological analysis and evaluation of the immune response.

#### Pathological analysis

The tumors and myocardium of the treated mice were embedded in paraffin after being fixed with paraformaldehyde. Images were obtained with a fluorescence microscope (Olympus BX53, Japan) for pathological analysis after the slices of tissues were stained with H&E.

#### T cell expansion

Lymphocytes were extracted from the collected tumors, lymph nodes, and spleens from the treated mice with the Lymphocyte Separation Kits (Beijing Solarbio Science & Technology Co., China), and then stained with fluorescence-labeled anti-CD3, CD4, and CD8 antibodies. A flow cytometer (FCM) was used to detect the percentage of T-cell subsets in each group.

#### Cytokine secretion

2.5 × 10^5^ mouse splenic lymphocytes from treated mice co-cultured with DOX-treated E.G7 cell debris for 72 h. The supernatant was collected after centrifugation at 450 g to measure cytokines IFN-α, IFN-γ, IL-12, and IL-1β using ELISA kits.

#### Memory T cells

Mouse splenic lymphocytes were seeded in a 24-well plate and co-cultured with E.G7 cell debris for 72 h. The lymphocytes were collected and stained with anti-CD4, CD44, and CD62L antibodies. The proportion of memory T cells was detected with FCM.

#### Lymphocyte proliferation after re-stimulated with antigens

Mouse splenic lymphocytes were labeled with anti-CD3, CD4, CD8, and Ki67 antibodies after being re-stimulated with E.G7 cell debris for 48 h, then T cell proliferation was evaluated with FCM.

### Statistical analysis

The experimental data were expressed as mean ± standard deviation (SD). Student’s t-test and ANOVA were used to detect the statistical differences among experimental groups. It was considered that there was a significant difference when the P value is less than 0.05.

## Supplementary Information

**Additional file 1: Table S1. **The size, PDI and zeta potential of MCMD NPs in different solution. **Figure S1. **The size distribution of MCMD NPs in water. **Figure S2. **The expression levels of MHC-II on BMDCs treated with PBS, free MC and MC NPs were measured with flow cytometers. Data were expressed in the form of mean ± SD. * P<0.05, ****P<0.0001. **Figure S3. **The expression levels of CD80 on BMDCs treated with PBS, free MC and MC NPs were measured with flow cytometers. Data were expressed in the form of mean ± SD. ** P<0.01, ****P<0.0001. **Figure S5. **H&E staining results of tumor and heart tissues in different groups. **Figure S6.** Representative FACS plots and histogram of percentage of CD3^+^ CD4^+^ and CD3^+^CD8^+^ T cells in lymph nodes.

## Data Availability

All data generated or analyzed during this study are included in this article and the Additional Information. The additional file is available.
