# Supplementary material for: Tumor microenvironment-responsive spherical nucleic acid nanoparticles for enhanced chemo-immunotherapy
Source: J Nanobiotechnology. 2023 May 26;21:171. doi: 10.1186/s12951-023-01916-0 (PMC10214549; doi:10.1186/s12951-023-01916-0)
Supplement: Supplementary file 1 — Additional file 1: Table S1. The size, PDI and zeta potential of MCMD NPs in different solution. Figure S1. The size distribution of MCMD NPs in water. Figure S2. The expression levels of MHC-II on BMDCs treated with PBS, free MC and MC NPs were measured with flow cytometers. Data were expressed in the form of mean ± SD. * P<0.05, ****P<0.0001. Figure S3. The expression levels of CD80 on BMDCs treated with PBS, free MC and MC NPs were measured with flow cytometers. Data were expressed in the form of mean ± SD. ** P<0.01, ****P<0.0001. Figure S5. H&E staining results of tumor and heart tissues in different groups. Figure S6. Representative FACS plots and histogram of percentage of CD3+ CD4+ and CD3+CD8+ T cells in lymph nodes. [file 12951_2023_1916_MOESM1_ESM.docx]

Supporting Information

Tumor microenvironment-responsive spherical nucleic acid nanoparticles for enhanced chemo-immunotherapy

*Bing Ma,^1,#^ Yingying Ma,^1,#^ Bo Deng,^1^ Pengjun Xiao,^1^ Pengyu Huang,^1^ Dali Wang,^2,*^ and Lanxia Liu^1,*^*

^1^Tianjin Key Laboratory of Biomedical Materials, Key Laboratory of Biomaterials and Nanotechnology for Cancer Immunotherapy, Institute of Biomedical Engineering, Chinese Academy of Medical Sciences & Peking Union Medical College, Tianjin 300192, P. R. China.

^2^School of Chemistry and Chemical Engineering, Zhang Jiang Institute for Advanced Study, Shanghai Jiao Tong University, 800 Dongchuan Road, Shanghai 200240, P. R. China.

*Corresponding author

Dali Wang, Ph.D.

E-mail: energywang@sjtu.edu.cn

Lanxia Liu, Ph.D.

E-mail: liulanxiabme@163.com

**Table S1.** The size, PDI and Zeta potential of MCMD NPs in different solution.


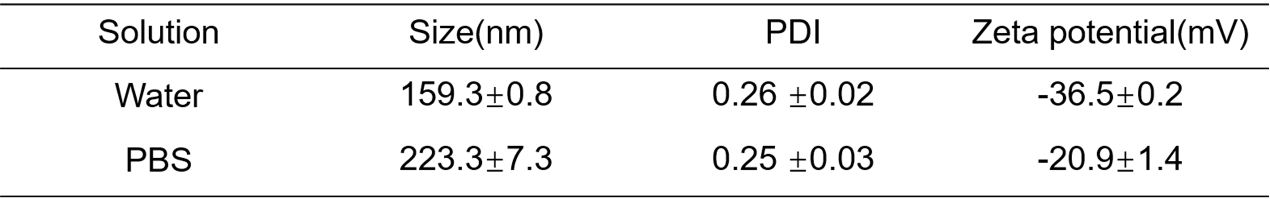
Data were expressed in the form of mean ± SD (n = 3)

**
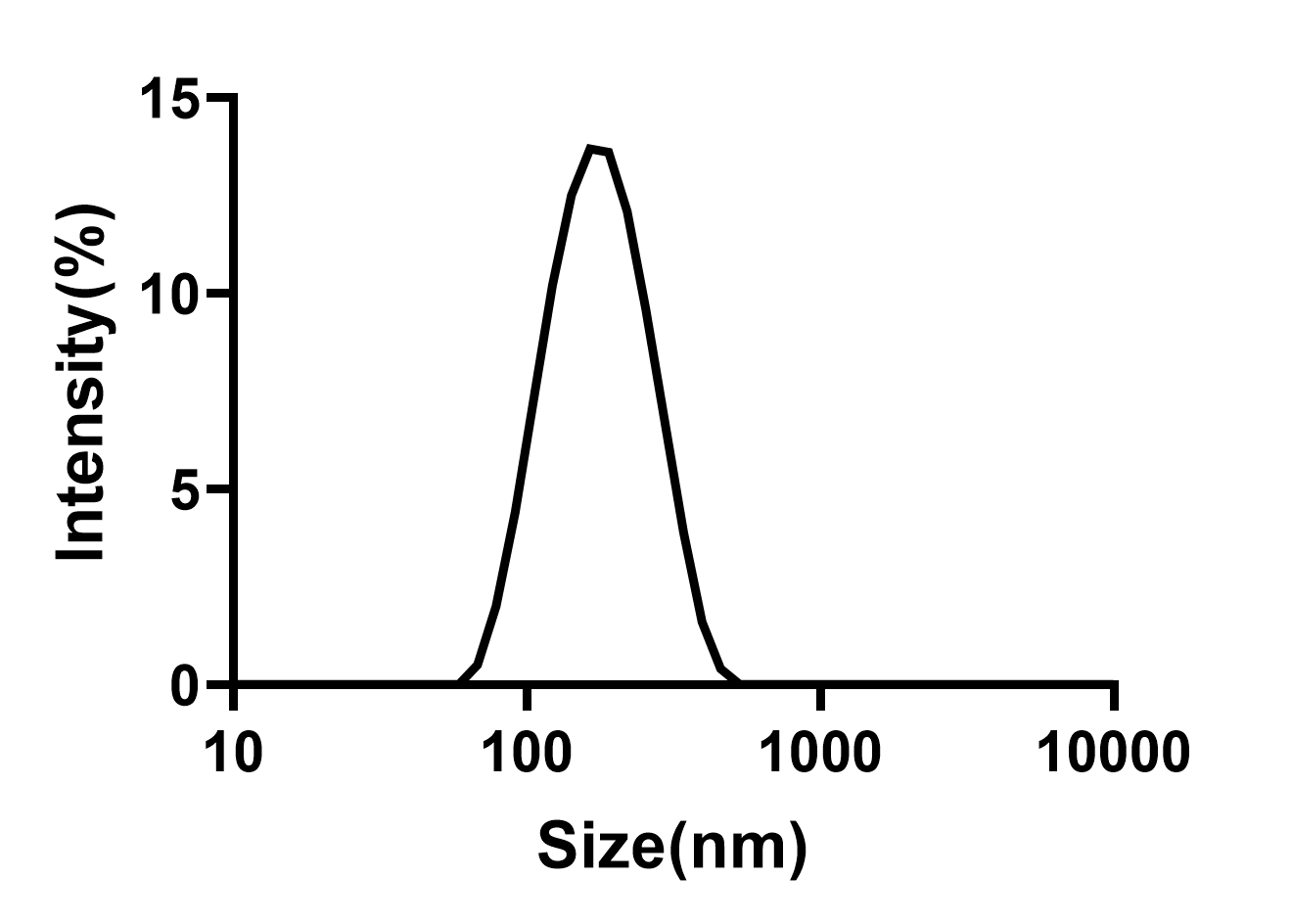
**

**Figure S1.** The size distribution of MCMD NPs in water.


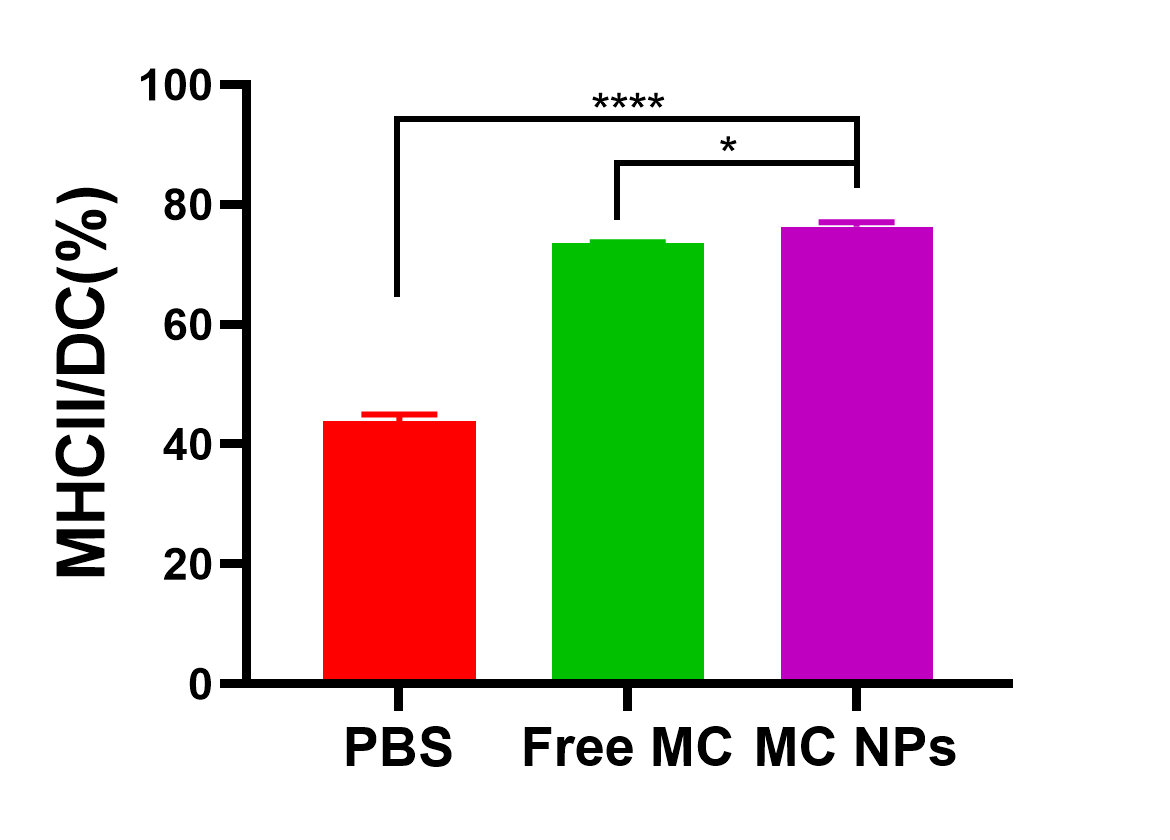


**Figure S2.** The expression levels of MHC-II on BMDCs treated with PBS, Free MC and MC NPs were measured with flow cytometers. Data were expressed in the form of mean ± SD (n = 5). * P<0.05, ****P<0.0001.


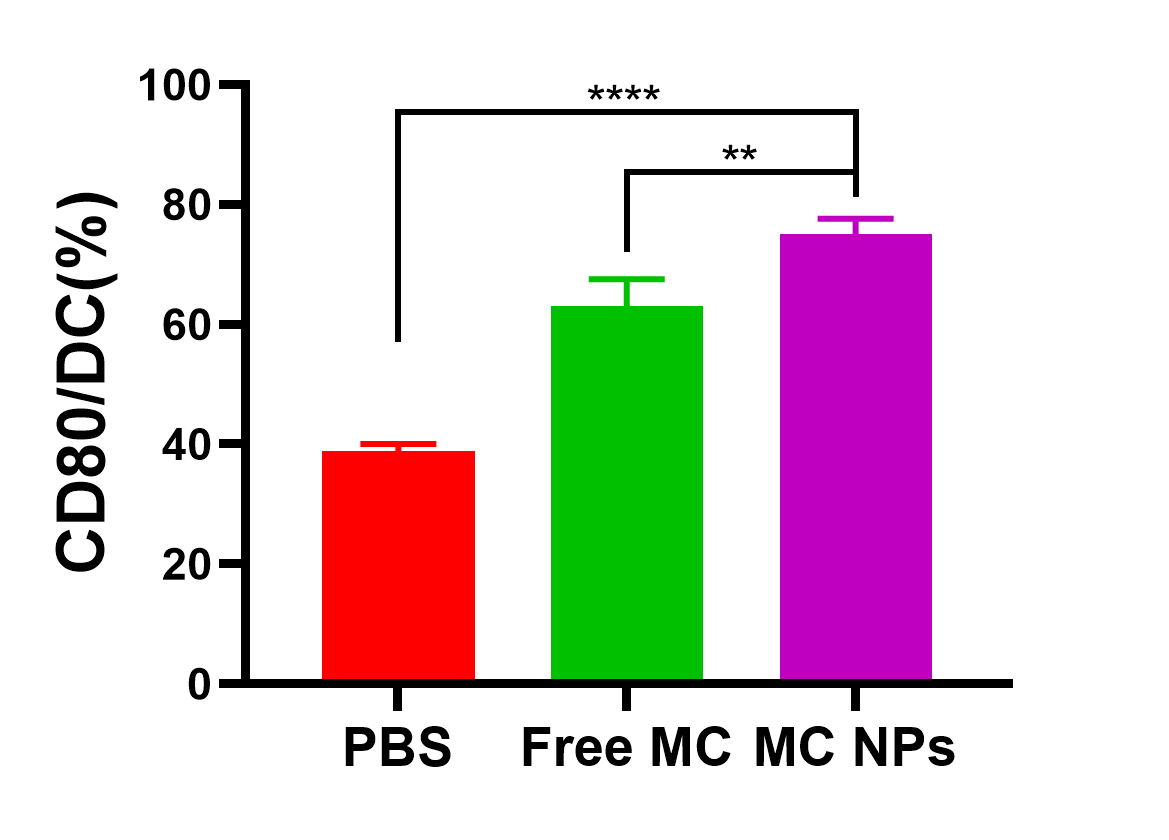


**Figure S3.** The expression levels of CD80 on BMDCs treated with PBS, Free MC and MC NPs were measured with flow cytometers. Data were expressed in the form of mean ± SD (n = 5). ** P<0.01, ****P<0.0001.


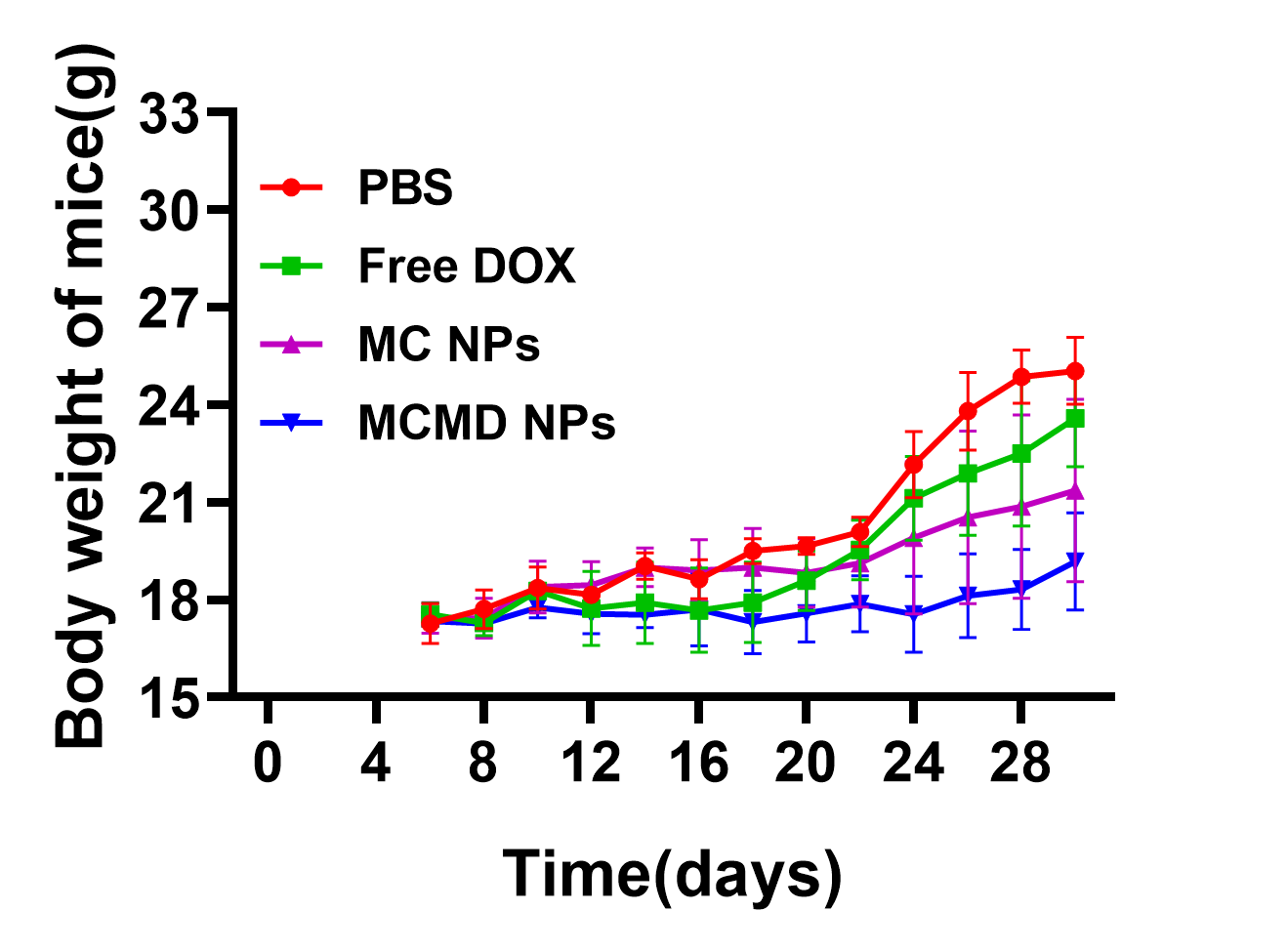


**Figure S4.** The body weight curves of mice treated with different formulations. Data were expressed in the form of mean ± SD (n = 5).

**
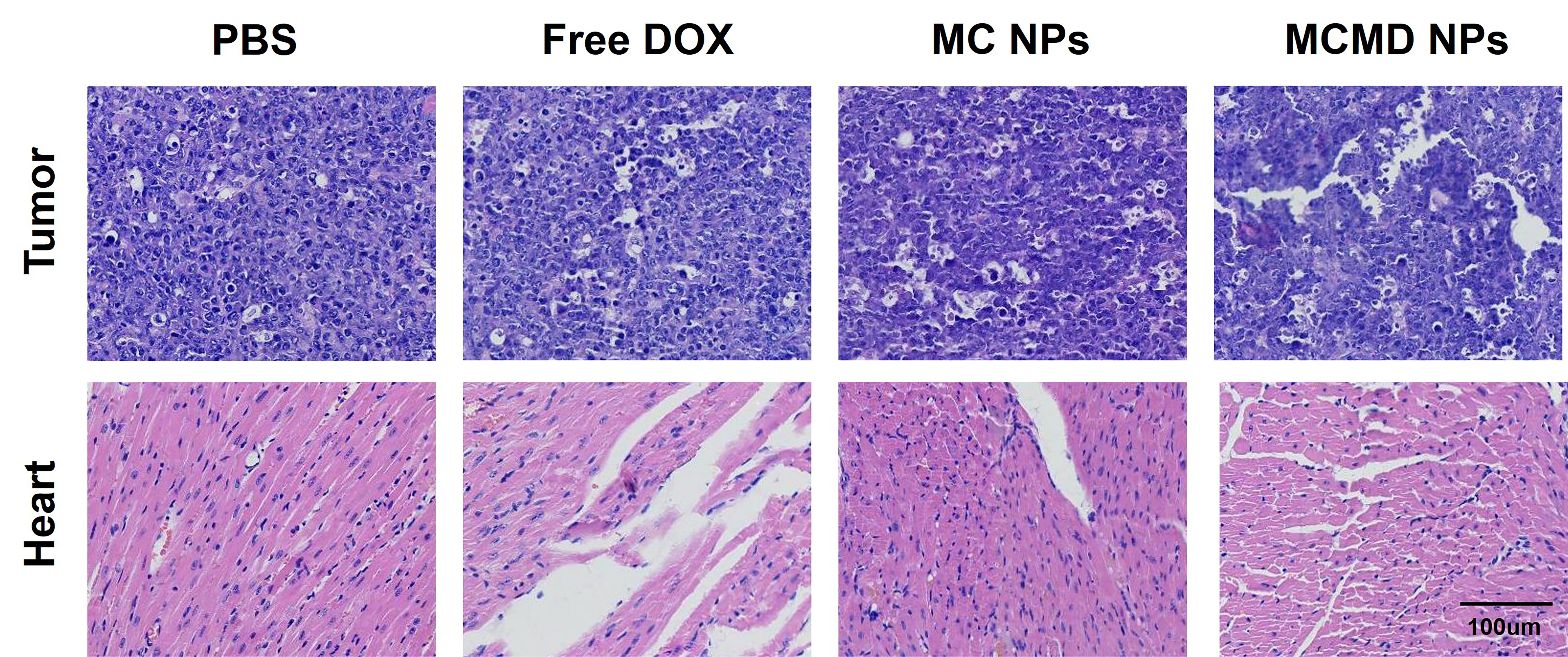
**

**Figure S5.** H&E staining results of tumor and heart tissues in different groups.

**
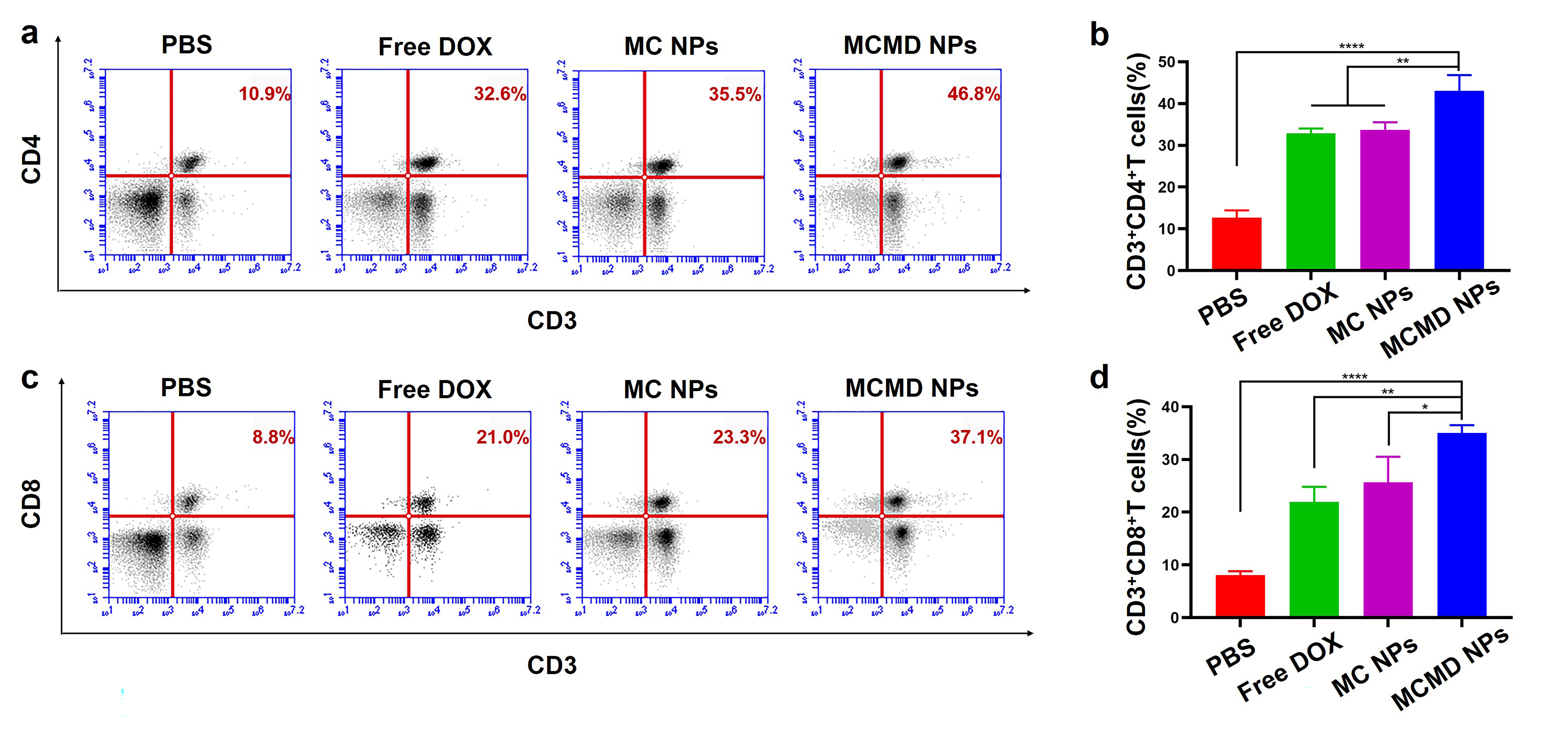
Figure S6.** Representative FACS plots and histogram of percentage of CD3^+^ CD4^+^ and CD3^+^CD8^+^ T cells in lymph nodes (A, B: CD3^+^CD4^+^; C, D: CD3^+^CD8^+^).
